# Multimodal AI-approach for the automatic screening of cardiovascular diseases based on nocturnal physiological signals

**DOI:** 10.1038/s44325-025-00051-z

**Published:** 2025-05-06

**Authors:** Youngtae Kim, Tae Gwan Jang, So Yeon Park, Ha Young Park, Ji Ae Lee, Tumenbat Oyun-Erdene, Sang-Ha Kim, Young Jun Park, Sung Pil Cho, Junghwan Park, Dongwon Kang, Erdenebayar Urtnasan

**Affiliations:** 1 Artificial Intellenge Big Data Medical Center, Wonju College of Medicine, Yonsei University Mirae Campus, Wonju, 26426 Republic of Korea; 2 Division of Pulmonary, Allergy, and Critical Care Medicine, Department of Internal Medicine, Wonju Severance Christian Hospital, Wonju, 26426 Republic of Korea; 3 Division of Cardiology, Department of Internal Medicine, Wonju Severance Christian Hospital, Wonju, 26426 Republic of Korea; 4 MEZOO Co., Ltd, Wonju, Republic of Korea; 5 MEDIANA Co., Ltd, Wonju, Republic of Korea; 6 Department of AI Semiconductor, Yonsei University Mirae Campus, Wonju, 26493 Republic of Korea

**Keywords:** Cardiology, Diseases

## Abstract

This study proposes a multimodal AI algorithm called the SleepCVD-Net to automatically screen CVDs based on nocturnal physiological recordings. We designed and implemented a multimodal AI algorithm, SleepCVD-Net, which utilizes three-mode deep neural networks to process input signals—single-lead electrocardiography (ECG), Airflow, and oxygen saturation (SpO_2_). Nocturnal physiological recordings were extracted from 194 subjects (80 controls and 114 subjects with CVD) in the Sleep Heart Health Study database. The proposed SleepCVD-Net model demonstrated good performance, achieving a mean accuracy of 97.55% on the test set. The F1-scores were 97.97%, 96.35%, 97.79%, and 97.49% for the control, stroke, angina, and congestive heart failure groups, respectively. The results indicate the potential for the automatic screening of CVDs based on nocturnal physiological signals. Furthermore, the SleepCVD-Net can serve as a valuable tool for monitoring cardiac activity during sleep in inpatient, outpatient, and home healthcare settings.

## Introduction

Cardiovascular diseases (CVDs) encompass various heart and blood vessel disorders, and they represent the leading cause of mortality worldwide, responsible for 32% of all global deaths, equating to 17.9 million fatalities in 2019^1^. This category includes conditions, such as coronary artery disease, which can precipitate chest pain, myocardial infarction, or stroke, as well as congestive heart failure, congenital heart disease, arrhythmias, and endocarditis^2^. Additionally, CVDs can serve as a risk factor for chronic conditions, such as chronic kidney disease^3^, obesity^4^, hypertension^5^, and diabetes mellitus^6^, alongside other adverse health states, such as physical inactivity^7^. Consequently, the early detection and prescreening of CVDs is imperative from clinical, social, and economic perspectives.

Polysomnography (PSG) is the gold standard for diagnosing and monitoring sleep disorders, including insomnia^8^, parasomnia^9^, hypersomnolence^10^, sleep-related breathing^11^ and movement disorders^12^, and circadian rhythm disorders^13^. PSG provides comprehensive physiological data, encompassing electroencephalography, electrooculography, electromyography, electrocardiography (ECG), oxygen saturation (SpO_2_), Airflow, movement, and respiratory parameters^14^. We hypothesize that PSG is a huge and informative diagnostic tool, because its extensive diagnostic capabilities can be leveraged for diagnosing not only sleep disorders but also other chronic conditions when combined with advanced artificial intelligence (AI) models, functioning as a preventative or prescreening tool for comorbidities and risk factors.

Electrocardiography (ECG) is a critical bio-signal recording that captures essential features such as the QRS complex, RR interval, heart rate variability, motion artifacts, and respiration. ECG has been extensively utilized as the primary input in numerous studies focused on the detection, classification, and diagnosis of CVDs, utilizing variations in heart rate variability parameters from single-lead^15,16^, 12-lead^17^, and portable/Holter ECG devices^18^. Similarly, medical imaging modalities, such as computed tomography^19^, magnetic resonance imaging^20^, and other imaging technologies^21^ are pivotal in CVD detection^22,23^. These bio-signals and medical images are measured during the daytime and in the awake state of the subject. To the best of our knowledge, no conventional studies have used nocturnal data to screen or detect CVDs. Furthermore, nocturnal physiological signals and PSG have never been previously used for the prescreening and early detection of CVDs, even though humans spend a third of their lifetime during sleep.

Many outstanding methods were proposed for CVD screening and detection in the last decade. These methods can be categorized by the model architecture, including single model, combined model, and multimodal-based approaches. Many of the conventional studies employed the single model-based method for CVD screening by applying machine learning or deep learning techniques^17–19,21–23^. Some studies investigated the combined model-based approaches that mixed various methods and classifiers for CVD screening^20,24^. Recently, multimodal based models were introduced to automatically detect CVDs using diverse data sources^25–27^. Multimodal methods integrate heterogeneous data, including physiological signals, medical images, electronic health records, and clinical data, and concurrently analyze them for data abstraction and event detection. However, there are very few studies of the multi-in multi-out and end-to-end approaches for the automated screening of CVDs. Moreover, to date, a multimodal AI-algorithm has not been developed for automatically screening CVDs utilizing PSG data.

In this study, we introduce a multimodal AI-algorithm, termed SleepCVD-Net, designed for the automatic screening of CVDs using nocturnal physiological recordings. The SleepCVD-Net model employs three-mode custom-designed deep convolutional neural network (CNN) models to extract informative feature maps from each input signal. The Sleep Heart Health Study (SHHS) database was utilized for building a real clinical PSG dataset to develop and validate the SleepCVD-Net model.

## Results

**Fig. 1:**
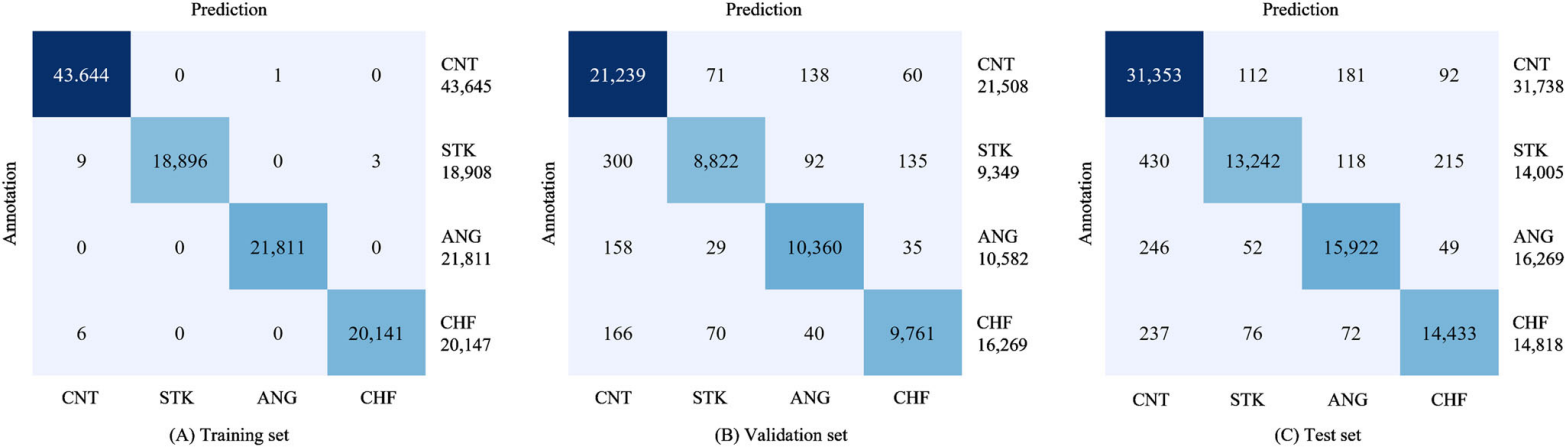
Confusion matrix of the proposed multimodal SleepCVD-Net for the CVD screening. A. Training set, B. Validation set, C. Test set.

**Fig. 2:**
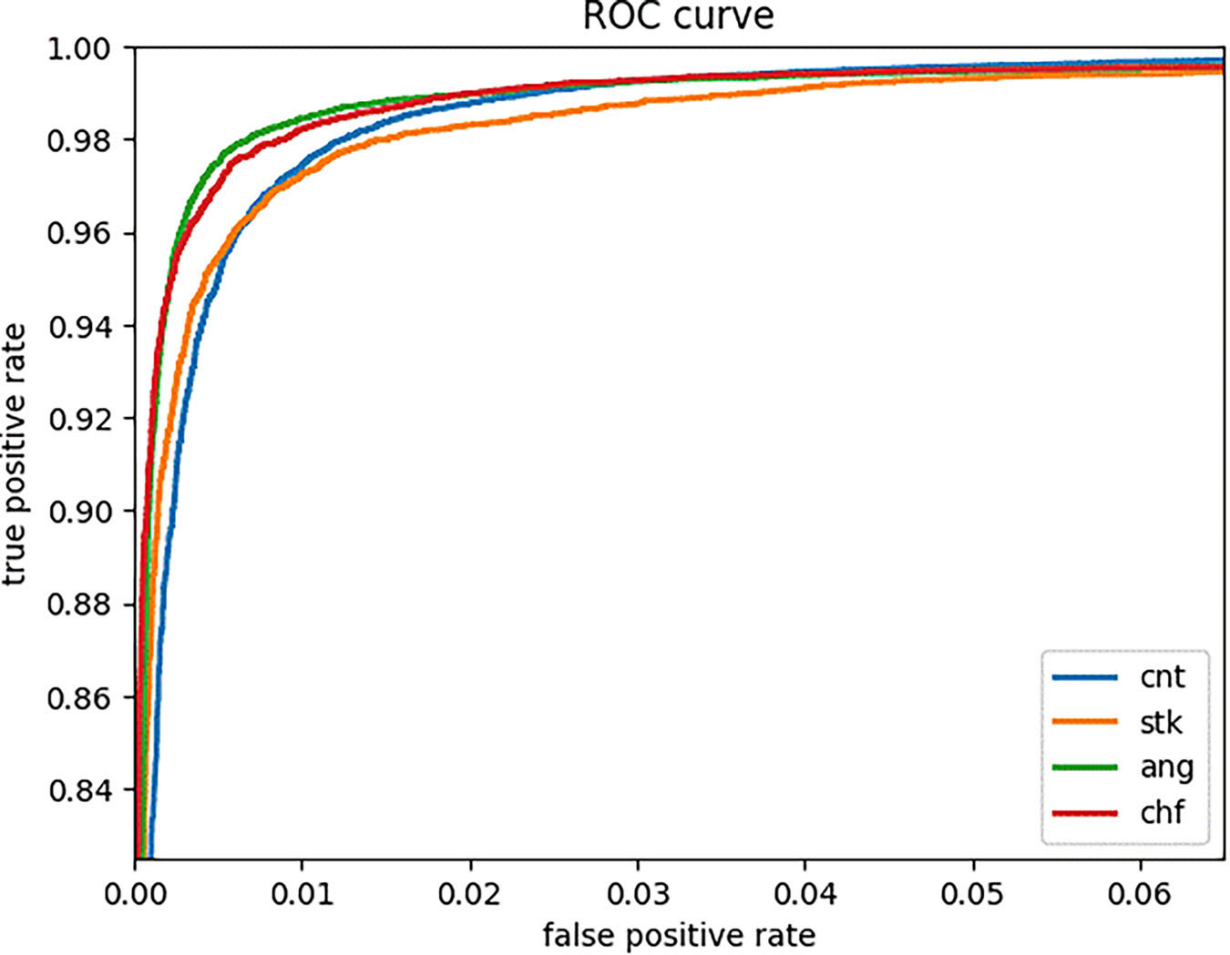
The receiver operating characteristic curve of the proposed multimodal SleepCVD-Net for the automatic screening of CVD based on nocturnal physiological recordings.

The performance of the SleepCVD-Net model is presented in three formats: a performance table (Table 1), confusion matrix (Fig. 1), and ROC curves for each class (Fig. 2). The proposed multimodal SleepCVD-Net model demonstrated robust and consistent performance across all CVD outcomes including healthy control (CNT), stroke (STK), angina (ANG), and congestive heart failure (CHF).

**Table 1:** Total performance of the multimodal SleepCVD-Net model

Datasets	Patients	Precision	Recall	F1-score	Accuracy
Training set	CNT	0.9996	0.9999	0.9998	0.9998
	STK	1.0000	0.9993	0.9996	
	ANG	0.9999	1.0000	0.9997	
	CHF	0.9998	0.9997	0.9997	
Validation set	CNT	0.9714	0.9874	0.9794	0.9748
	STK	0.9810	0.9436	0.9620	
	ANG	0.9746	0.9790	0.9768	
	CHF	0.9769	0.9725	0.9747	
Test set	CNT	0.9717	0.9878	0.9797	0.9755
	STK	0.9822	0.9455	0.9635	
	ANG	0.9772	0.9786	0.9779	
	CHF	0.9759	0.9740	0.9749	

Note: *CNT* control, *STK* stroke, *ANG* angina, *CHF* congenital heart failure.

There was no significant difference in performance between the validation and test sets, demonstrating the stability and robustness of the SleepCVD-Net model. The total average accuracies of the SleepCVD-Net model were 99.98%, 97.48%, and 97.55% for the training, validation, and test sets, respectively (Table 1).

Confusion matrices were used to illustrate the agreement between the predictions of the proposed SleepCVD-Net model and the cardiologist’s diagnoses. Additionally, we sought to identify which classes exhibited the highest error rates among the CVD categories. The confusion matrices for the training set (Fig. 1A), validation set (Fig. 1B), and test set (Fig. 1C) displayed similar patterns. Notably, the confusion matrices for the validation and test sets demonstrated stable predictions.

Finally, we assessed the false positive rate of the proposed SleepCVD-Net model for each target class: control, stroke, angina, and congestive heart failure. The receiver operating characteristic curves for the test set are presented in Fig. 2, demonstrating high performance for each CVD class.

## Discussion

In this study, we propose the SleepCVD-Net model, a multimodal AI algorithm designed for the automatic screening of CVDs using nocturnal physiological signals. The SleepCVD-Net employs a three-mode AI algorithm, comprising Mode_ECG, Mode_Airflow, and Mode_SpO2, each corresponding to single-lead ECG, Airflow, and SpO2 signals, respectively. These modes represent the cardiorespiratory activity and characteristics of the subjects. The model demonstrated very high performance, achieving an average accuracy of 97.55% for the target CVDs in the test set. Consequently, the proposed multimodal AI algorithm can be utilized as an extension tool to support CVD screening in conventional PSG studies for sleep monitoring and screening.

Several studies have explored various methods for detecting or predicting CVDs using single- or multi-lead ECG signals. Some recent studies are analyzed and listed in Table 2 for comparison with the methods and performance of our study. Most conventional studies based on physiological signals utilized daytime single-lead ECG, two-lead ECG, and standard 12-lead signals for the automated detection of CVDs and abnormal cardiac rhythms. Therefore, it may not be appropriate to directly compare the performance of this study with these studies. However, our results show comparable or similar performances than all these conventional studies (Table 2).

**Table 2:** Performance comparison with other studies

Study	Year	Sensors	Method	Accuracy	Sensitivity	Specificity
Dai et al. ^16^	2022	12-lead ECG	CNN	99.59	99.04	99.87
Wang et al. ^17^	2021	Single-lead ECG	Rule-based	90.47	81.59	91.75
Desai et al. ^28^	2017	Single-lead ECG	Ensemble learning	99.51	98.82	99.83
Pławiak^18^	2017	Single-lead ECG	SVM	98.85	-	99.39
Bagheri et al. ^25^	2020	Medical text, data	biLSTM	84.70	83.8	-
Li et al. ^26^	2021	ECG and PCG	SVM	87.40	90.30	84.50
Pan et al.^27^	2022	12-lead ECG	MCA-net	93.65	96.55	88.59
Our method	2023	Single-lead ECG, Airflow, SpO_2_	SleepCVD-Net	97.55	97.14	-

Specifically, Dai et al.^16^ proposed a deep CNN model to automatically classify five different CVDs based on standard 12-lead ECG signals. They developed a CNN model for classifying 1, 2, and 3-s ECG signals, achieving the highest accuracy, sensitivity, and specificity with 1-s ECG segments. Wang et al.^17^ introduced a rule-based algorithm for the automatic detection of abnormal heart rhythms from single-lead ECG signals developing and implementing 12 different rules for 11 types of abnormal ECG rhythms. Their method achieved an overall accuracy of 92.41% for detecting 12 different abnormal cardiac rhythms. Pławiak^18^ demonstrated an efficient multiclass classification method for 17 classes of ECG abnormalities using an evolutionary neural system and traditional machine learning methodologies, including ECG signal preprocessing, normalization, spectrum-based feature extraction, genetic optimization-based feature selection, and four different classifiers. The best performance, with a sensitivity of 90.2%, was achieved using a support vector machine. Desai et al.^28^ introduced an ensemble learning algorithm for automatically detecting myocardial ischemia from single-lead ECG signals. They extracted non-linear features from the ECG signal such as high-ordered statistics. They applied them to a decision tree, Adaboost, and Random Forest algorithms for myocardial ischemia detection with a very high-performance accuracy of 99.51%.

Recently, several studies have applied multimodal architectures for predicting CVDs and abnormal rhythm analysis. Bagheri et al.^26^ demonstrated a multimodal deep learning approach for CVD risk prediction using electronic health records. Their study incorporated heterogeneous data, including unstructured medical text records and structured clinical information. Various deep learning models were designed and evaluated, with the multimodal bidirectional long short-term memory model achieving the highest performance, marked by an F1-score of 83.8%. Li et al.^27^ proposed a machine learning-based multimodal approach for CVD prediction using physiological signals, such as single-lead ECG and phonocardiograms. They designed a dual-mode deep learning model to extract feature vectors, which were then integrated using a genetic algorithm. The final CVD prediction was performed using a support vector machine, resulting in a notable performance with an F1-score of 87.4%. Pan et al.^29^ introduced a multitask channel attention network to localize and detect myocardial infarction using 12-lead ECG data. They employed a residual-based channel attention network to capture and integrate feature maps from different leads. Their model was trained and evaluated on two different open ECG databases, achieving a detection accuracy of 93.65% on the PTBXL dataset. In summary, these multimodal studies have proposed innovative methods and demonstrated robust performances for their target outcomes, indicating that multimodal approaches are becoming comprehensive solutions for complex medical diagnostics and predictions.

Clinically, the main finding of this study is that the proposed SleepCVD-Net model can effectively screen patients for CVDs, including stroke, angina, and congestive heart failure using nocturnal physiological signals. Additionally, it can extend the application of traditional diagnostic PSG, which is primarily used for sleep disorders such as sleep apnea, insomnia, and other parasomnias, to include diverse CVD screenings. The proposed SleepCVD-Net model is applicable not only to full nocturnal PSG but also to portable home PSG devices, enabling automatic CVD screening. This novel approach for detecting and screening CVDs during sleep has the potential to reduce the economic burden associated with CVD treatments and save time compared to conventional diagnostic methods.

From an engineering perspective, SleepCVD-Net was designed using a multimodal architecture to simultaneously process multi-signal inputs from PSG recordings using AI technology. The input signals, such as ECG, Airflow, and oximetry, have different data shapes and sampling rates. The model was custom-designed and optimized both as a whole and in its sub-modes for the automatic screening of CVDs from nocturnal physiological signals. Multimodal AI technology can extend and provide novel applications for traditional diagnostic and screening tools widely used in clinical settings, whether in hospitals or home healthcare environments.

However, this study had some limitations. The target CVDs were limited to stroke, angina, and congestive heart failure; future studies will focus on a broader range of CVDs. Additionally, clinical phenotypes and characteristics were not considered as model inputs. Finally, the proposed model requires significant computational resources to execute and obtain results. Future research will aim to address these limitations and provide more comprehensive solutions.

In summary, we propose the SleepCVD-Net model, which is based on a multimodal AI algorithm for the automatic screening of CVDs using nocturnal physiological signals. SleepCVD-Net employs a three-mode AI algorithm and utilizes three primary nocturnal physiological signals: single-lead ECG, Airflow, and SpO2, which capture the cardiorespiratory characteristics of the subjects. The model demonstrated excellent performance, achieving an average accuracy of 97.55% on the test set. Consequently, SleepCVD-Net can serve as an extension tool for conventional PSG studies by enhancing sleep monitoring and screening capabilities.

## Methods

### Study design

**Fig. 3:**
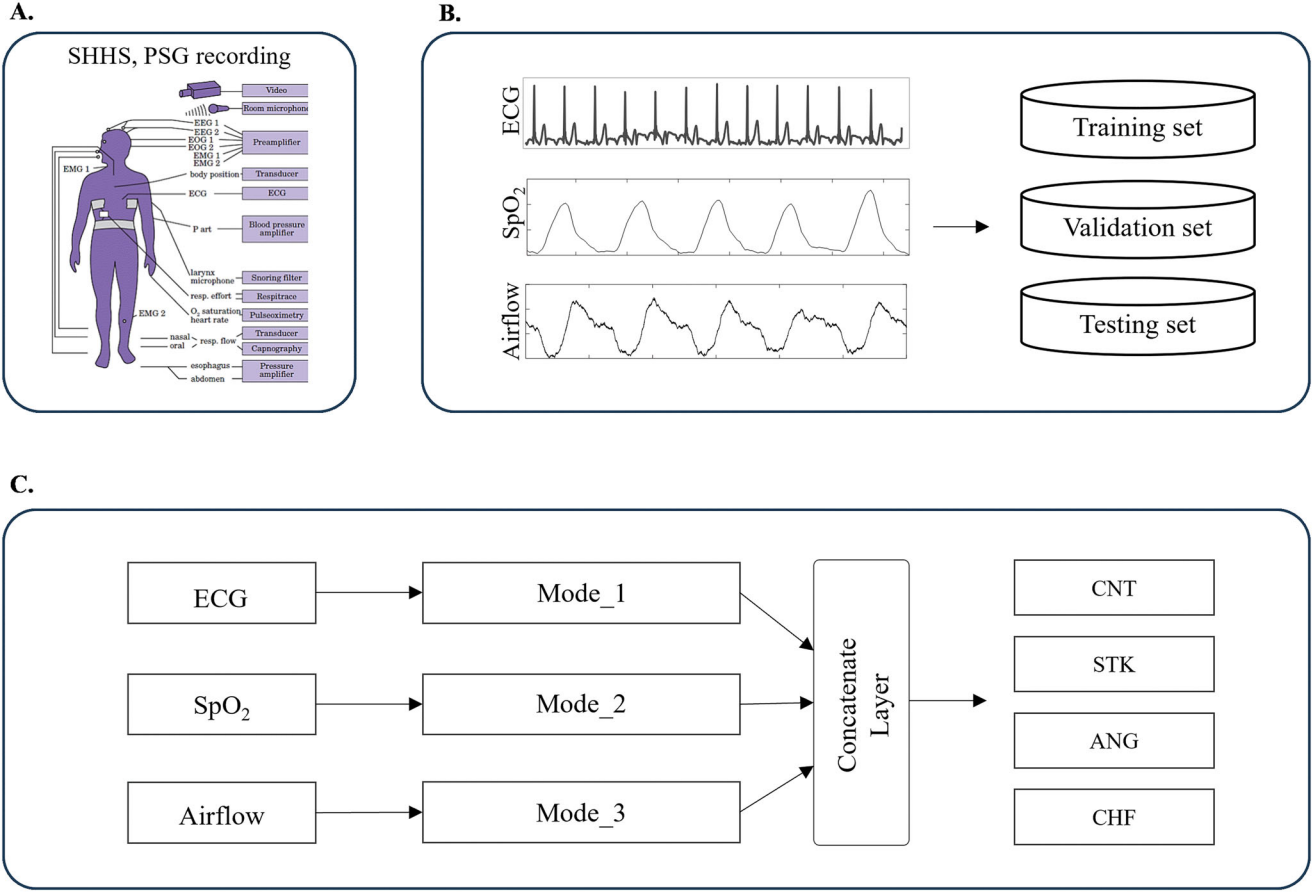
Study design of the proposed multimodal SleepCVD-Net model for the automatic screening of CVDs based on PSG recordings. **A** SHHS database that we analyzed for this study. **B** Extraction of PSG recordings and building of the physiological signal dataset. **C** Graphical overview of the proposed multimodal SleepCVD-Net model for automatic screening of CVDs.

The proposed multimodal SleepCVD-Net for the automatic screening of CVDs from PSG recordings comprises three primary components: a PSG study, nocturnal physiological signal datasets, and a multimodal AI-algorithm. A multicenter PSG cohort sleep study was conducted to recruit participants and obtain PSG datasets for the development and validation of the multimodal AI-algorithm (Fig. 3A). The three-channel nocturnal physiological signals were extracted from the PSG recordings to build the AI datasets (Fig. 3B). A multimodal AI-algorithm comprising three distinct sub-modes was designed and optimized for the automatic screening of patients with CVDs (Fig. 3C). A comprehensive description of each component is provided below.

### Study population

For the study population, data from the SHHS was utilized, and a multicenter cohort study was conducted by the National Heart, Lung, and Blood Institute to explore the association between cardiovascular outcomes and sleep-disordered breathing^29^. The SHHS was the pioneering study that investigated the relationship between sleep-related breathing disorders and the increased risk of CVDs and all-cause mortality. During the study, nocturnal full-PSG was performed on two occasions: during the first visit (November 1995 to January 1998) with 6441 participants, and during the second visit (January 2001 to June 2003) with 3295 participants. We utilized PSG data from all participants healthy control (CNT) group and diagnosed with stroke (STK), angina (ANG), and congestive heart failure (CHF); as these conditions were highly prevalent at the second visit stage of the SHHS study. All subjects provided written informed consents, and the study protocol was approved by The Institutional Review Board of the National Sleep Research Resources (sleepdata.org/datasets/shhs, IRB: NCT00005275).

We matched the control group with the CVD group from the same stage of the SHHS study at an approximate ratio of 2:1. In addition, we excluded the participants without PSG recordings from the data analysis. All 194 subjects (80 CNT and 114 CVD) who underwent a full-nocturnal PSG recording during the second visit of the SHHS were included in this study (Table 3). The baseline characteristics of the study population revealed significant differences in age, sleep efficiency, and total sleep time between groups. The demographic data indicated that the CVD group was older (mean age: 73.7 y), had poorer sleep quality (mean sleep efficiency: 74.9%), and shorter total sleep time (mean: 355.9 min) compared to the healthy control group. Our primary interest was in examining the nocturnal physiological signal differences between the CNT and CVD groups.

**Table 3:** Demographic characteristics of the study population

Measures	Total	CNT	CVD	*p*-value
Subjects (*N*)	194 (100.0)	80 (41.2)	114 (58.8)	0.04
Sex				
Male	95 (49.0)	32 (40.0)	63 (55.3)	*=*
Female	99 (51.0)	48 (60.0)	51 (44.7)	=
BMI (kg/m^2^)	28.06 ± 4.76	27.94 ± 4.56	28.14 ± 4.91	0.77
AHI (per h)	16.44 ± 16.11	14.16 ± 13.78	18.05 ± 17.44	0.10
Age (y)	69.92 ± 10.96	64.49 ± 11.21	73.73 ± 9.04	0.00
Race				0.23
White	169 (89.1)	69 (86.3)	102 (89.5)	=
Black	18 (9.3)	9 (11.3)	9 (7.9)	=
Other	7 (3.6)	4 (5.0)	3 (2.6)	=
Blood pressure (mmHg)				
Systolic	127.61 ± 17.20	124.84 ± 13.30	129.57 ± 19.31	0.05
Diastolic	69.87 ± 10.34	71.17 ± 10.41	68.95 ± 10.23	0.14
Sleep efficiency (%)	77.72 ± 12.80	81.64 ± 9.03	74.97 ± 14.29	0.00
Total sleep time (min)	373.51 ± 72.32	398.56 ± 58.35	355.92 ± 76.13	0.00

Note: *N* Numbers, *BMI* Body mass index, *AHI* Apnea hypopnea index.

### Nocturnal physiological dataset

In this study, we constructed a nocturnal physiological dataset using ECG, SpO_2_, and Airflow signals extracted from full-PSG recordings. During PSG, nocturnal ECG signals were recorded using Ag/AgCl electrodes at a sampling rate of 250 Hz. SpO2 was measured with Nonin XPOD 3011 and 8000 sensors at a 1 Hz sampling rate, and Airflow signals were obtained using a Compumedics thermistor at a 10 Hz sampling rate. For preprocessing, a high-pass filter (0.05 Hz) was applied to both the nocturnal ECG and Airflow signals at the hardware level ^30^.

All three physiological signals were extracted from the entire duration of the PSG recordings, excluding the initial and final 15 min to eliminate the wake or noisy sections. Following data extraction, the average total sleep time was 373.51 min. This data was segmented into 30-s events without overlap for the nocturnal physiological channels. The resulting nocturnal physiological dataset was then used to develop and evaluate the multimodal SleepCVD-Net model. To do this, the study population was randomly selected percent of 70 for the development group and 30 percent for the evaluation group. Ten percent of the development data set was used as validation set in training phase. The dataset was divided into a training set (104,511 segments) consisting of 116 subjects, a validation set (51,476 segments) of about 20 subjects, and a test set (76,830 segments) composed of 58 subjects, as detailed in Table 4.

**Table 4:** Distribution of the nocturnal physiological dataset

Dataset	CNT	STK	ANG	CHF	Total
Training set	43,645	18,908	21,811	20,147	104,511
Validation set	21,508	9,349	10,582	10,037	51,476
Test set	31,738	14,005	16,269	14,818	76,830
Total	96,891	42,262	48,662	45,002	232,817

Note: *CNT* control, *STK* stroke, *ANG* angina, *CHF* congenital heart failure.

### SleepCVD-Net

The proposed multimodal AI-algorithm comprises three sub-modes, each with distinct structures and hyperparameters. The distinguished physiological signals-ECG, SpO2, and Airflow-differ significantly in sampling rates and the information they provide. As we aimed for the applicability of the multimodal AI-algorithm across various scenarios, including in-hospital full PSG and out-of-hospital portable PSG setups, we incorporated the three physiological signals from PSG recordings. Furthermore, our goal is for the multimodal AI-algorithm to expand the utility of the PSG traditionally used for diagnosing sleep disorders, including the detection of CVDs. This expansion would provide additional valuable information to sleep technicians and specialists. Detailed descriptions of the implementation are provided below.

#### A. Nocturnal physiological signals

**Fig. 4:**
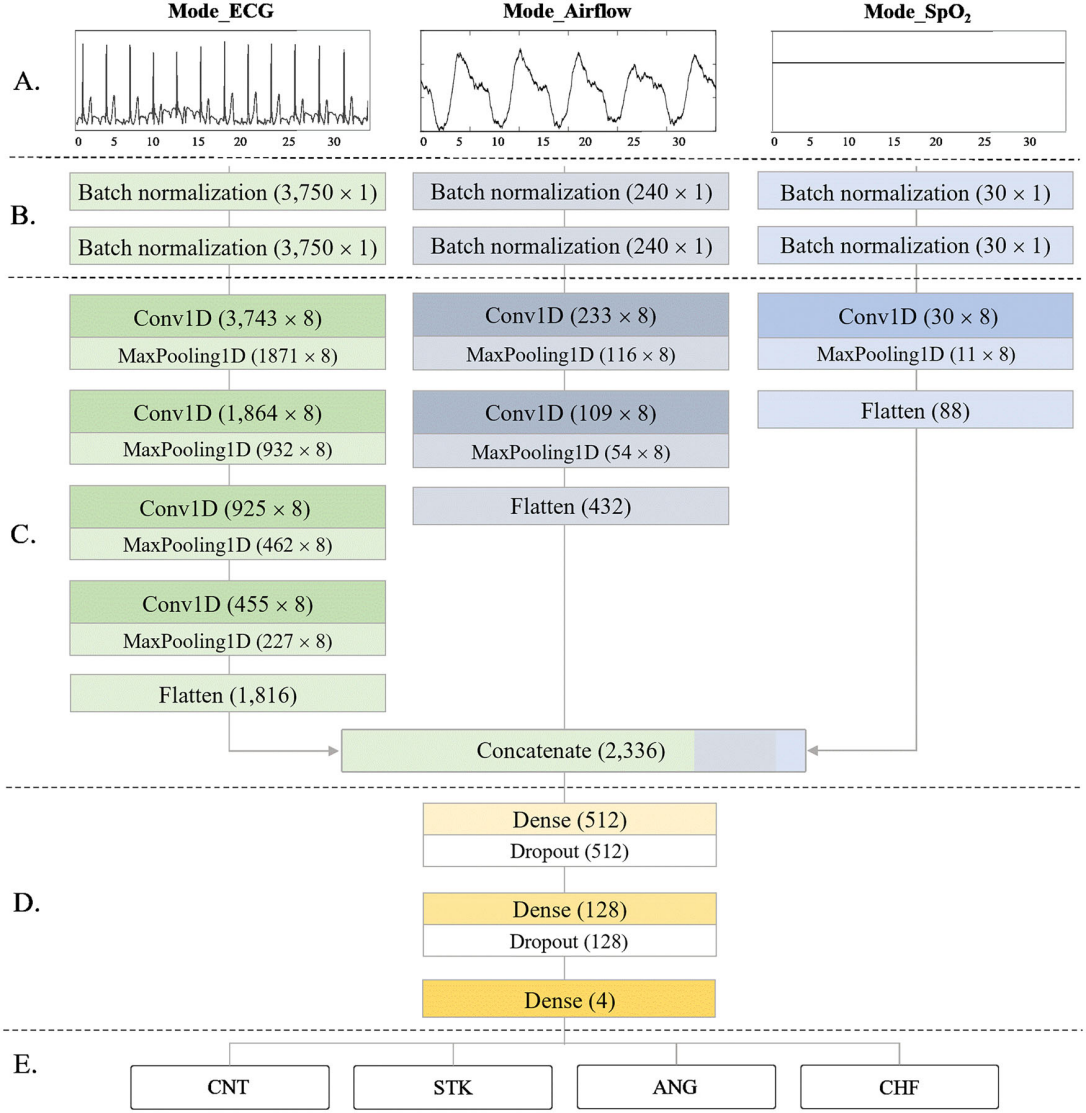
Graphical overview of the proposed SleepCVD-Net model for the automatic screening of CVDs based on nocturnal physiological recordings. **A** Multimodal input consisting of nocturnal physiological signals, including ECG, airflow, and SpO_2_. **B** Data preprocessing was performed for raw signal normalization using two-layer batch normalization for each modality. **C** Feature extraction was conducted to generate a comprehensive feature map by combining three sub-feature maps. **D** The classification network for final discrimination was constructed using a multilayer fully-connected neural network. **E** The proposed multimodal SleepCVD-Net model produced predictive outcomes based on nocturnal physiological recordings. Mode_ECG is a sub-mode for the single-lead ECG signal processing and feature map generation. Mode_Airflow is a sub-mode for the Airflow signal processing and feature map extraction. Mode_SpO_2_ is mode of the oxygen saturation signal processing and feature maps.

Three primary physiological signals were used as multi-input data for the multimodal AI-algorithm (Fig. 4A). Firstly, ECG was utilized to record the electrical signals of the heart rhythm for analyzing cardiac activity. ECG exhibits cardiac rhythms, which represent the periodic contraction and relaxation of the heart muscles. Through the characteristics of the R, P, and T waves from the QRS complex, we analyzed the heart rhythm and cardiac activity. The oxygen flow through the upper airway into the lungs were measured from Airflow. It monitors airflow limitation, reduction, and patterns, diagnosing respiratory issues, such as sleep apnea and hypopnea. By analyzing the cycle, the depth, regularity of inhalation and exhalation, and respiratory patterns were assessed. Finally, the oxygen saturation in the blood was measure using SpO_2_, which indicate how efficiently the blood transports oxygen; a range of 95–100% is typically considered healthy. By analyzing this data, the cardiovascular health can be determined through the patterns of the heart and respiration.

The ECG measures heart rhythm, Airflow assesses respiratory patterns, and SpO_2_ gauges oxygen saturation, collectively evaluating the overall health. Distinguished nocturnal signals were directly input into the model without any preliminary feature extraction or signal processing. The input shapes for the modes were 3,750 × 1 for ECG, 240 × 1 for Airflow, and 30 × 1 for SpO2 signals, respectively.

#### B. Data pre-processing

After signal selection, data preprocessing is essential for each bio-signal in both machine learning and deep learning models. Distinguished nocturnal signals (ECG, Airflow, and SpO_2_) may include various noises due to external interference or the signal-capturing process. Noise can degrade the data quality, thereby reducing the accuracy of model training and prediction. Although raw biological signals contain a wealth of meaningful information, they may be difficult to use directly in models. If significant features are not extracted, the model may fail to learn meaningful patterns from the biological signals. Biological signals may also contain measurement errors or sudden outliers, which can hinder model training and result in performance degradation. Through preprocessing, biological signal data can be refined and made more consistent, enhancing model performance and contributing to more reliable prediction outcomes. Preprocessing was incorporated into the initial layers of the model, where batch normalization was applied twice to the inputs (Fig. 4B). This preprocessing step effectively handled signal processing tasks, such as detrending and correcting baseline wandering. Theoretically, batch normalization can be represented by Eq. 1.

1 $${x}_{b}=\alpha \cdot \left(\frac{{x}_{i}-\mu }{\sqrt{{\sigma }^{2}+\varepsilon }}\right)+\beta$$

where *ε* is a random noise, *μ* is the mean of the mini-batch, *σ* is the variance of the mini-batch, *α* is a scaler, and *β* is a shift parameter. Both *α* and *β* are trainable and updated in an epoch-wise manner ^31^.

#### C. Feature map nets

At this stage, high-dimensional data abstraction was performed to extract informative feature maps. We employed deep CNN models with four-layer, two-layer, and one-layer structures for Mode_ECG, Mode_Airflow, and Mode_SpO2, respectively (Fig. 4C). The ECG, Airflow, and SpO2 are all time-series data that vary over time. A 1D CNN uses filters that move along the time axis to effectively learn patterns in time-series data. This approach is advantageous for capturing local patterns in time-series data and extracting features. Given that time-series data is represented as one-dimensional arrays, using 1D CNN results in fewer parameters compared to a 2D CNN; making the training process faster and more memory-efficient. Biological signals contain local patterns (e.g., the QRS complex in ECG, respiratory cycles in Airflow, and sudden fluctuations in SpO2). A 1D CNN can learn filters that effectively recognize these local patterns, highlighting important parts of the biological signals while removing unnecessary information. By using separate 1D CNN layers, the unique features of each signal can be independently extracted, allowing the model to learn significant patterns without interference between signals.

All convolutional layers were implemented using 1D convolution, which is suitable for analyzing time series data as it is simpler and faster than 2D convolutions. The 1D convolution operation can be represented as follows

2 $${x}_{k}={b}_{k}+\displaystyle \sum _{i=1}^{N}{w}_{k}\times {y}_{i}$$

where *x*_*k*_ is the *k*-th feature, *b*_*k*_ is the bias of the *k*-th feature, *w*_*k*_ is the *k*-th convolutional kernel from all features of the *k*-th feature, and *y*_*i*_ represents the *i*-th feature ^32^.

1D max-pooling layers follow all 1D convolutional layers to reduce the dimensions of the intermediate feature maps. This operation is known as 1D pooling when a 1D kernel is used. All pooling layers utilize maximum pooling. The proposed SleepCVD-Net network employs the rectified linear unit (ReLU) as the activation function in each layer, which can be represented as

3 $$f(x)=\max (0,{wx}+b)$$

[where *x* is the feature-map, *w* is the weight, and *b* is the bias]. The ReLU activation function provides robust training performance and consistent gradients, thereby facilitating effective gradient-based learning ^33^.

After feature extraction on each mode, feature map flattening was performed. The extracted feature map shapes were 1816 × 1 for Mode_ECG, 432 × 1 for Mode_Airflow, and 88 × 1 for Mode_SpO2. For the final decision-making process, all feature maps were concatenated, resulting in a final feature map shaped 2336 × 1.

Biological signals interact with each other, and for the diagnosis of cardiovascular diseases, it is important to comprehensively analyze various signals. By performing flattening followed by concatenation, features extracted from each signal are merged into a single vector, incorporating richer information. This allows the model to learn the correlations between multiple signals, enabling more accurate predictions. The flattening process converts high-dimensional features extracted by the CNN layers into 1D vectors. This helps to reduce the complexity of the model and increase computational efficiency. Concatenation integrates the features extracted from each input variable, enabling the model to learn the relationships between each input variable and make more accurate predictions based on these relationships. This process allows the unique characteristics of each input variable to be learned independently whilst modeling the complex interactions between them. It is particularly useful for dealing with complex problems where there are interactions between input variables.

#### D. Classification nets

Classification was done using a three-layer, fully connected network (Fig. 4D). This network was trained using concatenated feature maps to reach a conclusion regarding CVD classes. To optimize the classification performance and prevent overfitting and underfitting, dropout was employed. The specific structure of this section consisted of a dense_1 layer with 512 nodes, a dense_2 layer with 128 nodes, and a dense_3 layer with four nodes corresponding to each CVD class. Dropout was applied after each layer, and softmax activation was used in the final dense_3 layer to perform the final classification ^34^.

The concatenated vector is a large vector that includes various features. The dense layer helps to learn the interactions between the features within this large vector. This allows for a better understanding and discrimination of the comprehensive patterns in different biological signals. Dense layers (fully connected layers) enable the learning of more complex patterns by nonlinearly transforming the input features through numerous neurons. By combining the features extracted by the CNN layers and modeling various nonlinear relationships, they provide stronger representational power, enhancing the accuracy of detecting cardiovascular diseases. The dropout layer prevents overfitting by randomly deactivating neurons during the training process. By adding dropout layers, the model avoids excessive reliance on specific neurons or paths, improving generalization ability and performance on new data. This is crucial for maintaining robust performance across diverse data distributions, especially considering the variability and diversity of biological signal data. Adding dense and dropout layers twice ensures that the model undergoes a more stable and robust training process, reducing abrupt fluctuations in the loss function and allowing the model to learn in a smoother and more consistent manner. Dropout layers also help prevent overfitting, enabling the model to achieve more generalized performance.

#### E. CVD outcomes

The proposed multimodal SleepCVD-Net performed multiclass classification into four distinct CVD categories: control, stroke, angina, and congestive heart failure (Fig. 4E). A multiclass classification into four individual categories of CVDs is much more challenging than binary classification. Thus, for a more precise prediction of each category, the sub-mode must learn the unique patterns and maximum utilization of information obtainable from each signal—the ECG, Airflow, and SpO_2_. After separated feature map extraction, multiple signals and feature maps incorporate more information and the representational power of the proposed multimodal SleepCVD-Net model is enhanced, thereby improving the reliability and accuracy of cardiovascular disease detection. Learning the interactions and comprehensive patterns across multiple signals enables more sophisticated decision-making.

### Implementation

To implement the proposed SleepCVD-Net model, we employed a high-performance developing environment that entangled software with the Keras framework^35^ and a TensorFlow backend^36^. For model training and testing, an Intel CPU (i9-9900X @ 3.5 GHz) and NVIDIA GPU (GeForce RTX 3090) were used.

### Evaluation

The SleepCVD-Net model was evaluated using the accuracy and F1-score to assess the performance of automatic classification in different classes. To calculate the F1-score, the precision and recall indexes should be represented for all classes. Additionally, total accuracy was calculated to facilitate performance comparison with other studies. These metrics are defined as follows:

4 $${Accuracy}=({\rm{TP}}+{\rm{TN}})/({\rm{TP}}+{\rm{TN}}+{\rm{FP}}+{\rm{FN}})$$

5 $${Precision}={\rm{TP}}/({\rm{TP}}+{\rm{FP}})$$

6 $${Recall}={\rm{TP}}/({\rm{TP}}+{\rm{FN}})$$

where TP, FP, TN and FN denote the number of true positive, false positive, true negative, and false negative events, respectively.

7 $$F1-{score}=\sum _{i}2\cdot {w}_{i}\frac{{precisio}{n}_{i}\cdot {recal}{l}_{i}}{{precisio}{n}_{i}+{recal}{l}_{i}}$$

where *i* is the class index; *w*_*i*_ = *n*_*i*_/*N* is the proportion of samples in class *i*; *n*_*i*_ is the number of samples in the *i*th class; and *N* is the total number of samples.

A confusion matrix is a tool used to evaluate a classification model by matching the actual classes with the predicted classes. By comparing the predicted classes with the actual classes, the confusion matrix is utilized to visually understand the performance of the model. The confusion matrix helps identify the types of errors the model makes more frequently. This insight was used to determine the necessary steps for improving the model performance. Thus, the confusion matrix was used to individually evaluate the performance of each class in a multiclass classification problem, aiding in the understanding of the model performance for each class.

The receiver operating characteristic (ROC) curve is a graph that shows the performance of a classification model across all classification thresholds, depicting the relationship between sensitivity and specificity. When the dataset is imbalanced, a simple accuracy metric can misrepresent the model performance. The ROC curve is less sensitive to class imbalance and can separately evaluate the classification performance of positive and negative classes. This makes it useful for assessing the true performance of a model on imbalanced datasets. The ROC curve uses the area under the curve (AUC) to evaluate the model’s performance, with a larger AUC indicating a higher accuracy of classification.

## Data Availability

sleepdata.org/datasets/shhs, IRB: NCT00005275.
